# Turning the Tide on Falls: A Narrative Review Toward Safer Aging

**DOI:** 10.7759/cureus.93845

**Published:** 2025-10-04

**Authors:** Ramakant Yadav, Ajay Emani, Roopesh Kirar, Midhun Mohan

**Affiliations:** 1 Neurology, Uttar Pradesh University of Medical Sciences, Etawah, IND

**Keywords:** definition, dementia and fall, elderly, epilepsy and fall, etiology, falls, management, neurodegenerative diseases

## Abstract

Falls are a significant source of morbidity and mortality in the elderly, contributing to functional decline, loss of independence, and increased healthcare burden. Falls are an important public health problem that has a great effect on the quality of life of an individual.

Peer-reviewed original and systematic review articles were sourced, which were published from January 2000 until January 2025 in the PubMed/Medline, Embase, and Cochrane Library databases using keywords and Boolean operators like AND, or OR, such as "falls", "elderly", "definition", "management", "aetiology", "neurodegenerative diseases", "epilepsy and fall", "dementia and fall", and prevention and diagnostic tests for postural instability and falls.

This article integrates current guidelines and research to present a comprehensive review on falls in older adults, addressing epidemiology, pathophysiology, risk factors, consequences, and multifaceted prevention strategies to guide clinicians and researchers.

## Introduction and background

An often-quoted observation by Professor Bernard Isaacs reminds us of the devastating impact falls can have on older adults: “It takes a child one year to acquire independent movement and ten years to acquire independent mobility. An old person can lose both in one day” [1].

Falls are not only a leading cause of injury and hospitalization in adults over 65 years - occurring annually in up to 30% of this population - but also the second leading cause of mortality worldwide according to the WHO [2]. Nearly half of those aged over 80 experience a fall each year, and recurrence rates are high, with half of fallers experiencing another fall within 12 months [3,4]. Despite advances in understanding, the consequences of falls remain severe, emphasizing the need for comprehensive risk identification and management.

Evidence-based interventions have been introduced to prevent falls, and identifying the aetiology of falls is the most important step in their management. This systematic review aims to scrutinize falls, their early diagnosis, and protocols for their management, with identification of research gaps.

## Review

Search methodology

Articles that were published from January 2000 to January 2025 were searched in the databases of PubMed/Medline, Embase, as well as Cochrane Library. Use a combination of Medical Subject Headings (MeSH) terms, Emtree (Embase), and keywords focused on: Population: “elderly”, “older adults”, “aging”, “geriatric”; Topic: “falls”, “recurrent falls”, “fall prevention”, “fall risk”, “falls epidemiology”, “falls classification”; Specific subtopics: “neurodegenerative disorders”, “dementia”, “Parkinson’s disease”; Management and interventions: “fall management”, “exercise”, “multifactorial intervention”, “environmental modification”; Guidelines: “World Falls Organisation”, “falls guidelines”, “clinical practice guidelines”. Filters used were English language, only human subjects, and studies included reviews, observational studies, clinical trials, as well as randomized controlled trials (RCTs). Full-text assessment of selected articles was done for eligibility based on the PICOS criteria (Population, Intervention, Comparison, Outcomes, and Study design).

Inclusion criteria included reviews, original articles, RCTs, and guidelines with observational cross-sectional studies. Exclusion criteria comprised studies outside the time frame or not addressing specified outcomes, case control, cohort studies, letters to the editor, studies whose full-text was not available, or case reports. Initial search across all databases yielded 6300 total records. Duplicates were removed. Screening of titles and abstracts for relevance to falls in the elderly, excluding irrelevant studies (wrong population, non-falls topics, outside date range), was done. Full texts were retrieved and assessed for potentially relevant articles against eligibility criteria (PICOS). Articles that didn’t meet the criteria were excluded.

Full texts of the studies were assessed for eligibility using the same inclusion criteria by two reviewers independently (E.A. and R.Y.). Full-text studies that did not meet the inclusion criteria were excluded, and the reasons for exclusion are reported. During the process, any disagreements that arose between the two reviewers were resolved through discussion. If a consensus was not reached, then the other two reviewers (R.S. and M.M.) were involved.

The included studies were critically assessed independently by two reviewers (E.A. and R.Y.) using the standardized Joanna Briggs Institute (JBI) checklist for prevalence studies. All the studies, irrespective of their methodological quality, underwent data extraction and synthesis, where possible. Data extraction was done by two reviewers using a pre-developed and pre-tested data extraction tool (E.A. and R.Y.). Again, in cases of disagreement, the other two reviewers were involved.

**Figure 1:**
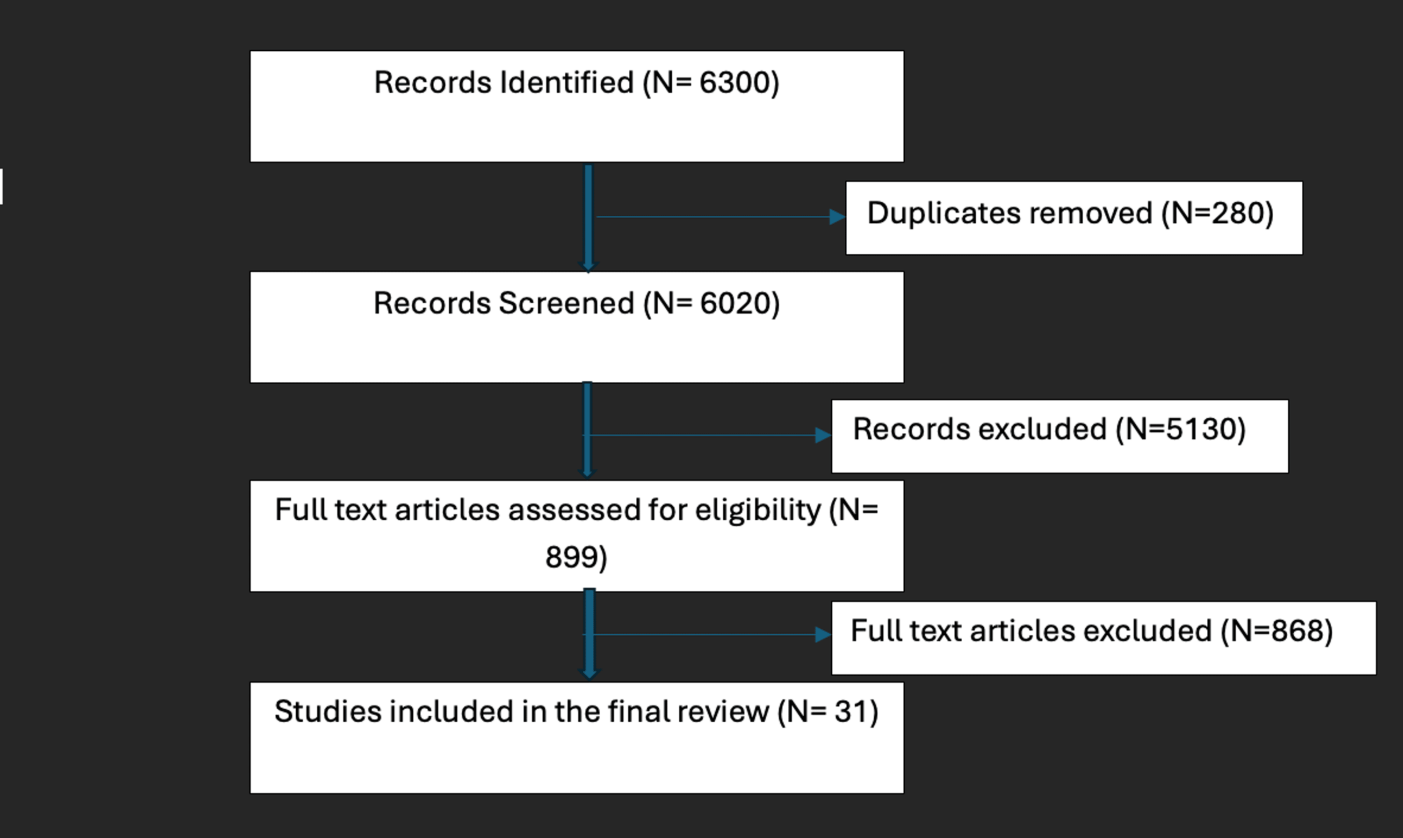
Preferred Reporting Items for Systematic Reviews and Meta-Analyses (PRISMA) flow chart

The search yielded 899 citations. Full-text and open-access articles were retrieved and reviewed. From these, 31 articles were included in our narrative review. Full-text articles that did not meet the eligibility criteria, involved the wrong population, or had language barriers were excluded. Figure 1 illustrates the Preferred Reporting Items for Systematic Reviews and Meta-Analyses (PRISMA) flow diagram for the studies identified and screened.

Defining falls: taxonomy and classification

A fall is defined as an event resulting in a person coming to rest unintentionally on the ground or another lower level, not as a result of a major intrinsic event (such as a stroke) or an overwhelming hazard [3]. Table 1 mentions the key terms used with falls.

**Table 1:** Falls and related terms Credit: Reference [4]

Term	Description
Falls	An unexpected event in which an individual comes to rest on the ground, floor, or a lower level.
Recurrent falls	Two or more falls reported in the previous 12 months.
Unexplained fall	When no apparent cause has been found for a fall on performing a multifactorial fall risk assessment, and it cannot be explained by a failure to adapt to an environmental hazard or by any other gait or balance abnormality.
Severe fall	Falls with injuries that are severe enough to require a consultation with a physician; result in the person lying on the ground without the capacity to get up for at least one hour; prompt a visit to the emergency room; associated with loss of consciousness.
Fall-related injury	An injury sustained following a fall.
Fall risk increasing drugs (FRIDs)	Medications known to increase the risk of falls.

The taxonomy of falls is categorized as extrinsic, intrinsic, non-bipedal, and non-classifiable [5]. Extrinsic factors are further classified as outlined in Table 2 [4].

**Table 2:** Extrinsic factors in falls Credit: Reference [4]

Category	Factor	Operational Definition
Slip	Slippery surface	Ice, snow, water, and objects can cause the legs to go out from under the patient.
Slip	Non-slippery surface	Icline, rough, uneven, and an object causing a perturbed stance.
Trip	Object or hazard	Discrete object on a flat surface.
Trip	Surface irregularity	Step, curb, and uneven sidewalk.
Displaced center of gravity	Inertial	Sudden acceleration (e.g., pulling a drawer that suddenly opens)
Displaced center of gravity	Collision	Being pushed or hit by a person or an object.
Displaced center of gravity	External load	Carrying a heavy object can cause initiation of a fall.
Displaced center of gravity	Unusual posture	Unusual position or motion, as in sport, avoidance behaviour where any fit individual would fall.

Table 3 presents the classification of intrinsic factors.

**Table 3:** Intrinsic factors in falls Credit: Reference [4]

Category	Factor	Operational Definition
Mobility systems failure	-	The leg gave out, muscle weakness, or other reasons, causing the inability to navigate.
Impaired balance	Vertigo	Sensation of spinning with or without association with a head movement.
Impaired balance	Head movement associated with a fall	Fall is associated only with a head movement, but no vertigo.
Impaired balance	Postural instability	Loss of balance during normal activity, with or without association with a postural change.
Sensory impairment	Visual	Visual problem initiating a fall
Sensory impairment	Other sensory	Hearing, poor sensation in the leg when asleep, initiating a fall.
Cognitive impairment	Global	Patient confused, delirious.
Cognitive impairment	Visuo-perceptual	Misperceiving the environment (e.g., unexpected surface, missing last step).
Cognitive impairment	Distraction	Sensory or internal distraction, not paying attention.
Loss of consciousness	-	Loss of consciousness not associated with a head movement or positional change.

Table 4 presents the categories of non-bipedal falls.

**Table 4:** Non-bipedal falls Credit: Reference [4]

Category	Factor	Operational Definition
Self-generated fall	Fall out of bed	Wake up on the floor, roll out of bed, or reach out of bed.
Self-generated fall	Other	Fall out of a chair or other, reaching out of the chair.
Support failure	Assistive device	Breaking, giving way.
Support failure	Furniture or other	The chair collapses, not involving movement initiated by the patient.

Table 5 presents the categories of non-classifiable falls.

**Table 5:** Non-classifiable falls Credit: Reference [4]

Category	Factor	Operational Definition
Unknown causes of falls	-	The interview was completed, but the patient could not describe the fall well.
Inadequate data	Patient deceased	No fall interview; patient, or collateral, unavailable, or unable to provide interview.
Inadequate data	Patient refused or dropped	-
Inadequate data	Unreliable or inadequate data	-

Discussion

Epidemiology and Consequences

One-third of people aged 65 or above fall each year, rising to nearly half for those over 80 [3]. More than half sustain repeated falls, and consequences can range from minor bruises to fractures, head injuries, loss of independence, or death. Falls also lead to psychological consequences (fear of falling, loss of confidence), social isolation, and increased caregiver burden.

Pathophysiology of Falls

Aging brings multisystem changes that heighten fall risk. One of these changes is sarcopenia, which results in decreased muscle strength and mass, further reducing stability [4,6]. The elderly are susceptible to neuromuscular dysfunction, thus affects balance, motor response, and coordination. The cognitive impairment that progressively occurs with age results in a slowed reaction and poor hazard recognition [4]. Usually, the prevalence of comorbidities is high with old age, such as diabetes, neurodegenerative disorders, and more, which impair the physiological reserve in the elderly. Obesity and sedentarism reduce mobility and magnify the risk of falls. The fragility of bone also increases with age, resulting in increased fracture risk after falls [4].

Risk Factors

Lord et al. provided a classification system for fall risk that consists of seven domains: mobility, psychological, environmental, medical, drug-associated, sociodemographic, and neuromuscular or sensory [7]. Four of these also carried the risk of recurrent falls: medication, mobility, psychological, and sensory, as well as neuromuscular abnormalities. Further risk factors that can cause falls are spinal cord disorders. Falls in an osteoporotic individual can be impactful [8]. Previous history of a fall is the most common risk factor [9]. Fractures that result from “low energy trauma”, that is, falling from a standing height or less, that usually would not result in a fracture, are called fragility fractures. Visual prowess is also weak in older adults, which impairs gait and balance control and increases fall risk [10-12].

Table 6 presents the classification of domains described by Lord et al.

**Table 6:** Risk factors: Lord's domains Credit: Reference [4]

Domain	Subtypes
Psychosocial and demographic	Advanced age, female gender, living alone, history of falls, inactivity, activities of daily living (ADLs), and alcohol intake
Mobility	Impaired stability while standing, impaired stability when leaning, inadequate responses to external perturbations, and slow stepping
Sensory and neuromuscular	Reduced visual acuity, reduced vestibular function, and muscular weakness
Medication	Psychoactive substance use, NSAID use, and antihypertensive use
Environmental	Poor footwear, inappropriate spectacles, home hazards, and external hazards
Medical	Stroke, Parkinson’s disease, vestibular disorders, decreased bone mineral density, and impaired cognition

Special Considerations: Neurodegenerative Diseases

Parkinson’s disease (PD): Most falls occur forward during movement. Freezing of gait, impaired proprioception, balance deficits, and medications all contribute [13]. Non-forward falls also occur, such as during sitting or standing. Freezing of gait typically results in forward falls, whereas balance impairments and the akinetic-rigid subtype are associated with backward falls. Backward falls are also observed in Pisa syndrome and camptocormia, conditions commonly linked with PD. Mechanisms include impaired interlimb coordination, postural instability due to abnormal processing of proprioceptive signals in the basal ganglia, and excessive activation of antagonist muscles when posturally perturbed, often in anxious states.

Progressive supranuclear palsy (PSP): Falls may be an early feature, often due to impaired locomotor pathways and brainstem nuclei dysfunction.

Alzheimer’s and other dementias: Environmental hazard risks are most frequently associated with falls in Alzheimer’s disease. Falls in dementia with Lewy bodies (DLB) are due to visuospatial, executive deficits, and orthostatic hypotension. Frontotemporal dementia (FTD) falls are linked to gait disturbance and cognitive impairment; vascular dementia is also associated with gait disturbance.

Multiple system atrophy, cerebellar ataxia, and epilepsy: Falls are frequent due to disequilibrium and cognitive or autonomic dysfunction.

Vision and falls: Normal vision makes a person safely negotiate stairs and steps [12]. Older adults tend to have reduced visual acuity and hence, are 1.7 times more likely to fall and 1.9 times more likely to have multiple falls as compared to fully sighted adults [14].

Consequences of a Fall

A spectrum of effects is seen once fall occurs in the elderly that can be broadly classified into physical, mental, and social categories. The physical effects include bruising, fractures, burns, intracranial hemorrhages, and death. Depression, fear, and a restriction of lifestyle are common among the mental consequences of a fall. Social consequences are the inability to leave home, the requirement of long-term care, and the inability to travel.

Assessment of Risk and Diagnostic Methods

Robust evaluation is crucial following a fall or in those at risk. In hospital settings, tools such as the Hendrich II Fall Risk Model and STRATIFY are commonly used. Among the community, the Timed Up and Go (TUG) test, the Three Minute Backward Walking (3MBW) test, the Chair Stand test, and multi-stage balance tests (parallel, tandem, and single-leg) are employed. Key physical assessments include postural hypotension, vision, cognition, and neurologic deficits. Gait, transfers, and the use of assistive devices need to be assessed. Medications known as fall risk-increasing drugs (FRIDs) should also be screened. 3MBW test at 3.5 seconds is a novel test with a specificity of 61% and a sensitivity of 74%, much higher than the TUG test [15]. Modifications of risk factors at home reduce the risk of falls by 38% [16].

**Figure 2:**
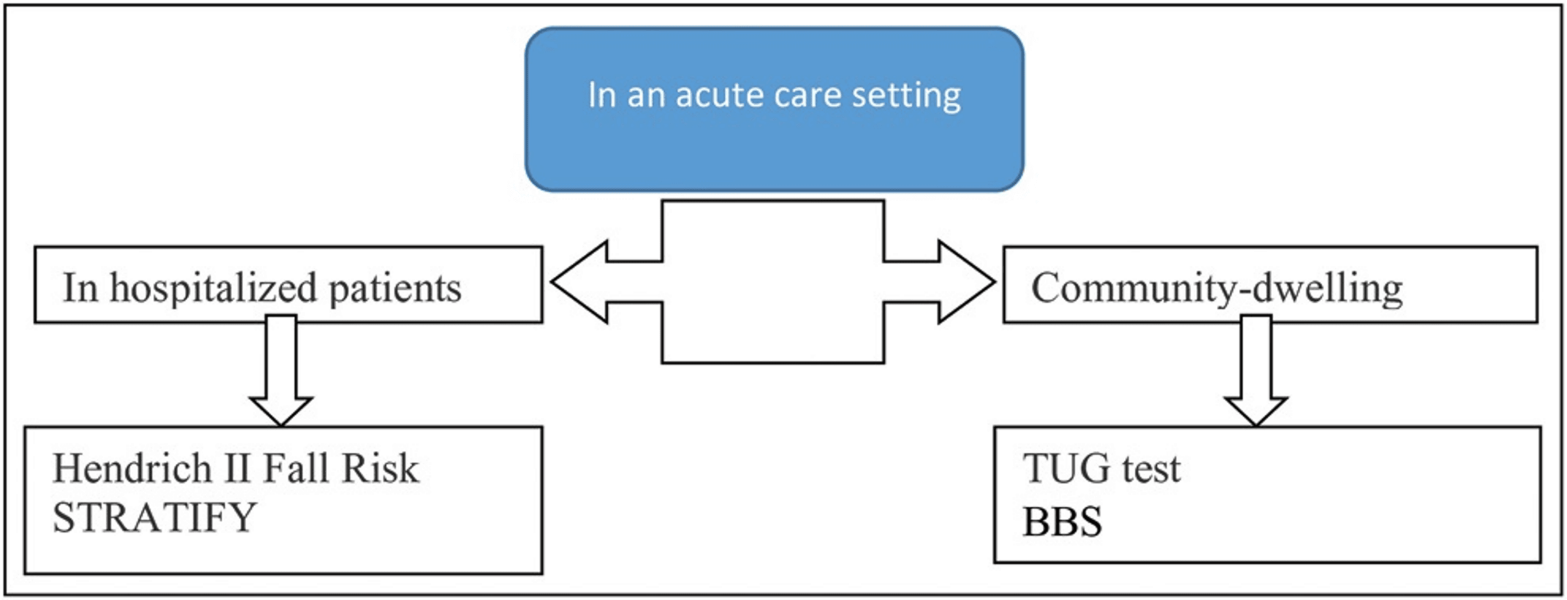
Assessment tools for a fall TUG: Timed Up and Go Test; BBS: Berg Balance Scale Credit: Reference [4]

**Figure 3:**
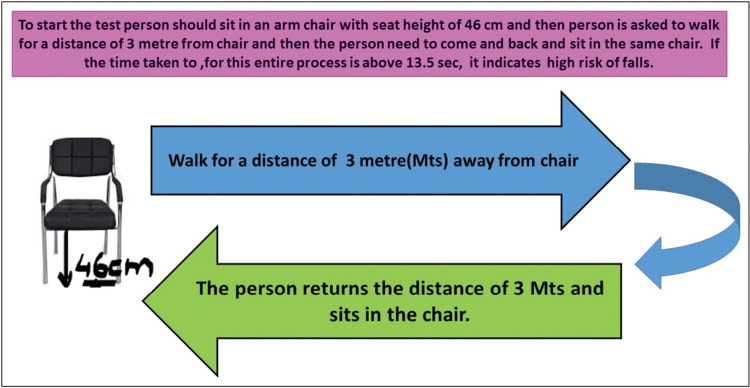
Timed Up and Go Test Credit: Reference [4]

Figures 2-3 depict the approach to evaluation in hospitalized patients and community-dwelling individuals, and the procedure for the TUG test, respectively.

Approach to Falls: A Multifactorial Strategy

**Figure 4:**
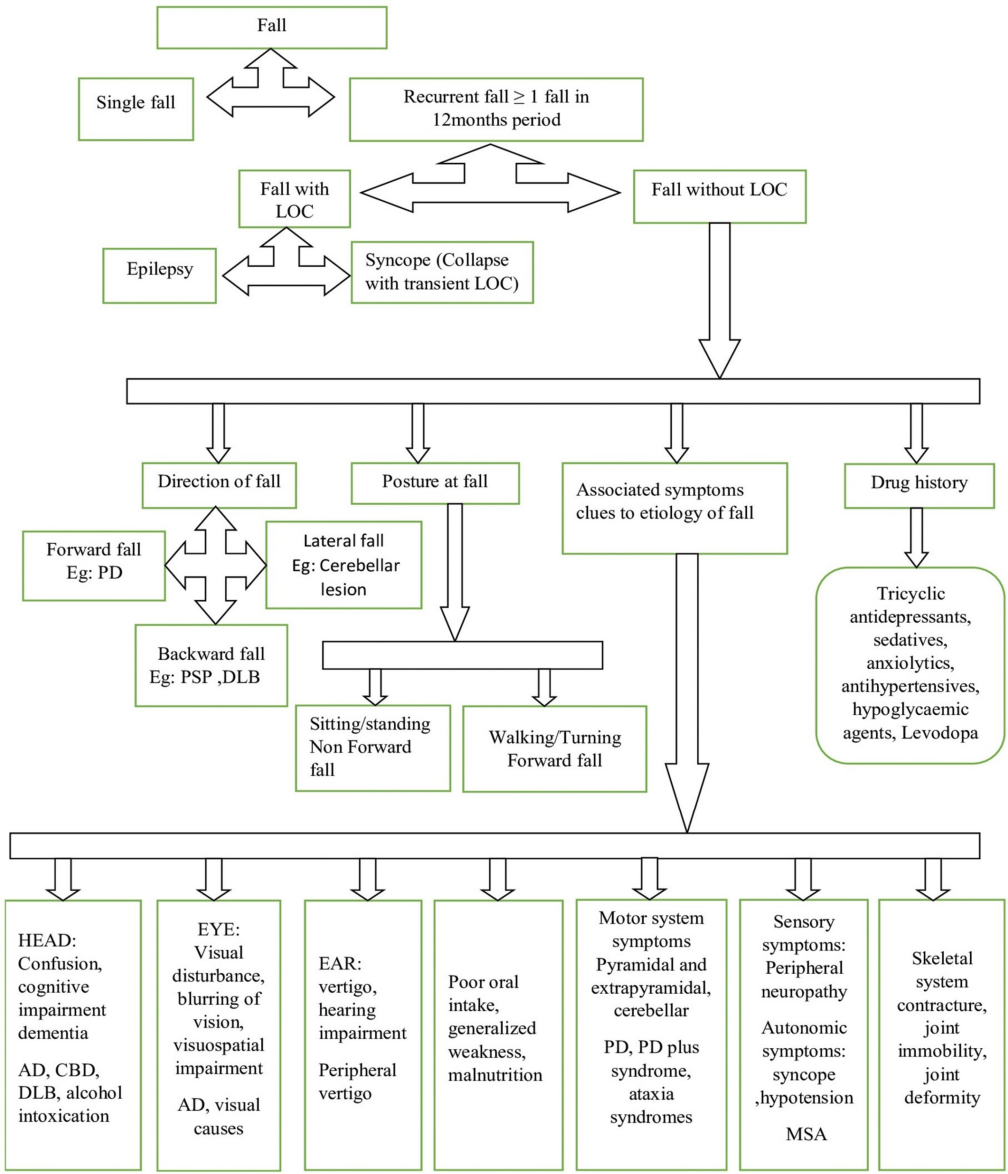
Approach to falls LOC: loss of consciousness; PD: Parkinson's disease; PSP: progressive supranuclear palsy; DLB: dementia with Lewy bodies; AD: Alzheimer's disease; CBD: corticobasal degeneration; MSA: multi-system atrophy Credit: Reference [4]

A comprehensive approach includes confirmation of single versus recurrent falls, screening for FRIDs and polypharmacy, the use of mnemonics (“CATASTROPHE” and “I HATE FALLING”) for systematic evaluation, and physical testing - minimum: TUG, Pull Test, and 3MBW (Figure 4). The elderly should be encouraged to engage in physical activity adapted to their abilities. Any presence of environmental and home hazards should be addressed. Underlying medical and neurodegenerative conditions need to be treated. A team-based approach should be promoted, including a neurologist, a geriatrician, an ophthalmologist, an orthopaedic, a physiotherapist, and caregiver involvement. A flow chart simplifying the approach to falls [4] can be used for proper management, as shown in Figure 4. Tables 7-8 elaborate on the full forms of the mnemonics "CATASTROPHE" and "I HATE FALLING", respectively.

**Table 7:** “CATASTROPHE” mnemonic for fall risk factors Credit: Reference [4]

Mnemonic Letter	Factor/Meaning
C	Caregiver and Housing Adequate?
A	Alcohol and alcohol withdrawal? Any Illicit drugs?
T	Treatment (medications and medication compliance)
A	Affect (depression)
S	Syncope
T	Teeing (dizziness and vertigo)
O	Ocular problems
P	Pain or problems with mobility
H	Hearing impairment
E	Environmental hazards

**Table 8:** “I HATE FALLING” mnemonic for fall risk factors Credit: Reference [4]

Mnemonic Letter	Factor/Meaning
I	Inflammation of Joints
H	Hypotension (orthostatic pressure changes)
A	Auditory and visual abnormalities
T	Tremors (Parkinson’s disease or other causes of tremor)
E	Equilibrium (balance) problem
F	Foot problems
A	Arrhythmia, heart block or valvular disease
L	Leg-length discrepancy
L	Lack of conditioning
I	Illness
N	Nutrition
G	Gait disturbances

Prevention and Guidelines (World Falls Guidelines)

Recent world guideline recommendations emphasize exercise, including balance, strength, and mobility regimens tailored to individual risk [17,18]. They also state the need for review of medications as well as deprescribing FRIDs where possible [19]. Vision correction [20] in the form of regular screening and appropriate correction plays an important role in the prevention of falls. Introduction of home safety interventions, such as removing tripping hazards, improving lighting, and installing grab bars, is necessary as per the guidelines [21-23]. Promoting bone health by screening and treating osteoporosis is also recommended [24,25]. Use of assistive devices [26-29] only after assessment of their appropriateness and safe use has been recommended by the guidelines. Broadly, a multidisciplinary care approach has been promoted to improve outcomes [30,31].

Limitations

The limitations of this review are that a detailed description of each component is beyond the scope of this article. Apart from that, it only included original articles, RCTs, and systematic reviews. Case reports, which may have described novel therapies, were not included.

## Conclusions

A pragmatic approach is proposed for the early detection of falls because of its increasing incidence. In this review, we discovered that the erstwhile definition of “recurrent falls” has changed from “two or more falls in a six-month duration” to “one or more falls in a 12-month duration”. Falls need to be evaluated in detail, including looking for sarcopenia, postural hypotension, assessment of vision, as well as cognitive function tests, in the case of recurrent falls. Falls in the elderly are multifactorial and require a systematic, interdisciplinary approach for effective prevention and management. Early risk identification, comprehensive assessment, and evidence-based interventions can reduce fall risk and its consequences, protecting independence and quality of life in older adults.
